# CYP2C19 Genotyping May Provide a Better Treatment Strategy when Administering Escitalopram in Chinese Population

**DOI:** 10.3389/fphar.2021.730461

**Published:** 2021-08-27

**Authors:** Xinyi Huang, Chao Li, Chaopeng Li, Zhenyu Li, Xiaohui Li, Jianwei Liao, Tai Rao, Lulu Chen, Lichen Gao, Dongsheng Ouyang

**Affiliations:** ^1^ Hunan Key Laboratory of Pharmacogenetics, Xiangya Hospital, Institute of Clinical Pharmacology, Central South University, Changsha, China; ^2^ Hunan Key Laboratory for Bioanalysis of Complex Matrix Samples, Changsha Duxact Biotech Co., Ltd., Changsha, China; ^3^ Hunan Changsha Duxact Clinical Laboratory Co., Ltd, Changsha Duxact Biotech Co., Ltd., Changsha, China; ^4^ Department of Geriatric Disorders, Xiangya Hospital, Central South University, Changsha, China; ^5^ National Clinical Research Center for Geriatric Disorders, Xiangya Hospital, Central South University, Changsha, China; ^6^ Department of Pharmacy, Cancer Institute, Phase Ⅰ Clinical Trial Centre, Changsha Central Hospital Affiliated to University of South China, Changsha, China

**Keywords:** selective serotonin reuptake inhibitor1, escitalopram2, pharmacogenomics3, individualized treatment4, clinical trials

## Abstract

Depression disorder is one of the most serious mental illnesses in the world. Escitalopram is the essential first-line medication for depression disorder. It is the substrate of hepatic cytochrome P450 (CYP) enzyme *CYP2C19* with high polymorphism. The effect of *CYP2C19* on pharmacokinetics and pharmacodynamics on Caucasian population has been studied. The Clinical Pharmacogenetics Implementation Consortium Guideline provides dosing recommendations for escitalopram on *CYP2C19* genotypes on the basis of the studies on Caucasian population. However, the gene frequency of the alleles of *CYP2C19* showed racial differences between Chinese and Caucasian populations. Representatively, the frequency of the *2 and *3 allele, which were considered as poor metabolizer, has been shown to be three times higher in Chinese than in Caucasians. In addition, the environments might also lead to different degrees of impacts on genotypes. Therefore, the guidelines based on the Caucasians may not be applicable to the Chinese, which induced the establishment of a guideline in China. It is necessary to provide the evidence of individual treatment of escitalopram in Chinese by studying the effect of *CYP2C19* genotypes on the pharmacokinetics parameters and steady-state concentration on Chinese. In this study, single-center, randomized, open-label, two-period, two-treatment crossover studies were performed. Ninety healthy Chinese subjects finished the trials, and they were included in the statistical analysis. The pharmacokinetics characteristics of different genotypes in Chinese were obtained. The results indicate that the poor metabolizer had higher exposure, and increased half-life than the extensive metabolizer and intermediate metabolite. The prediction of steady-state concentration based on the single dose trial on escitalopram shows that the poor metabolizer might have a higher steady-state concentration than the extensive metabolizer and intermediate metabolite in Chinese. The results indicate that the genetic testing before medication and the adjustment of escitalopram in the poor metabolizer should be considered in the clinical treatments in Chinese. The results provide the evidence of individual treatment of escitalopram in Chinese, which will be beneficial for the safer and more effective application of escitalopram in the Chinese population.

**Clinical Trial Registration**: identifier ChiCTR1900027226.

## Introduction

Depression disorder is a common mental disease that might lead to considerable difficulty with daily functioning (Cipriani et al., 2009). More seriously, the patients with severe depression showed a higher suicide rate than other mental diseases; the number of suicides from depression is estimated to reach 1 million each year worldwide (WHO Guidelines Approved by the Guidelines Review Committee, 2016). The World Health Organization (WHO) has warned depression disorder as a leading cause of disability around the world and significantly contributes to the global burden of disease (WHO Guidelines Approved by the Guidelines Review Committee, 2016). The situation of depression disorder in China is not optimistic either: At present, the incidence rate in China is about 3–5 percent, i.e., more than 36 million people suffer from depression disorder in China (Yu et al., 2021). Therefore, it is essential to treat and manage depression disorder.

Antidepressant is one of the key strategies to treat depression disorder (Sinyor, 2019). Escitalopram is an essential selective serotonin reuptake inhibitor (SSRI) antidepressant, which binds to the serotonin transporter protein (SERT) and inhibits the reuptake of serotonin by the presynaptic neuron (Stahl, 1998; Landy and Estevez, 2021). It is the (*S*)-enantiomer of citalopram, another SSRI antidepressant. Because the (*S*)-enantiomer of citalopram demonstrated significantly more potency than the (*R*)-enantiomer of citalopram relative to the serotonin reuptake and inhibition, escitalopram shows higher potency than citalopram (Rao, 2007). Therefore, it is widely used in the treatment of major depressive disorder and generalized anxiety disorder (Stahl, 1998; Landy and Estevez, 2021).

Escitalopram was developed under the trade name Lexapro^®^ by Lundbeck, Switzerland. It was launched in the United States in 2002, in China in 2006. Escitalopram is the substrate of hepatic cytochrome P450 (CYP) enzyme *CYP2C19*, which shows high polymorphism (Ji et al., 2014). *CYP2C19* exhibits 35 different types of gene alleles of gene polymorphism (PharmaVar, 2021). Among these gene alleles, the *1 allele is wild type with normal function; the *17 allele is the allele with increased enzyme activity; and the *2 and *3 alleles are the major variants (Ding et al., 2015). The effect of *CYP2C19* on the pharmacokinetics (PK) and pharmacodynamics (PD) on the Caucasian population has been studied: The subjects with the * 17 allele showed faster metabolisms, and the *2 and *3 alleles showed poorer metabolisms of escitalopram (Chang et al., 2014; Jukić et al., 2018). The Clinical Pharmacogenetics Implementation Consortium Guideline provides dosing recommendations for escitalopram on *CYP2C19* genotypes on the basis of the studies on Caucasian population (Hicks et al., 2015). However, *CYP2C19* shows different mutation rates in different races over the world: the frequency of the *2 and *3 alleles have been shown as 29–35% and 5–9% in Asians, whereas the frequency in Caucasians is 12–15% and <1%. The frequency of the *17 allele is reported to be higher in Caucasians (16–21%), but relatively lower (3–6%) in Asians (Dorji et al., 2019; Zhou et al., 2019). In addition, the environments may also lead to different degrees of impacts on genotypes. Therefore, the guidelines based on the Caucasians may not be applicable to the Chinese, which induced the establishment of a guideline in China. Escitalopram was approved for usage in China for 15 years, and it is the first-line antidepressant medication in China (Li et al., 2020). Therefore, it is necessary to evaluate the effect of *CYP2C19* genotypes on the PK parameters on Chinese population to provide a basis for the individualized medication guidance of escitalopram in China. For safer and more effective usage of escitalopram, the effect of food and gender on escitalopram also needs to be evaluated on Chinese populations (Chang et al., 2014; Jukić et al., 2018).

In this study, we evaluated the PK profiles of escitalopram under fasting and fed conditions in male and female subjects. Then, the effect of *CYP2C19* genotypes on the PK parameters of escitalopram was also evaluated. The steady-state blood concentration (C_ss_) of escitalopram was predicted from the PK parameters of different genotypes to provide the evidence to speculate as it is necessary to adjust the dose of escitalopram according to the genotypes in Chinese population.

## Subjects and Methods

### Subjects

Subjects were screened for eligibility approximately 1 week before dosing. Screening included medical history, vital signs, physical examination, clinical laboratory tests, and 12-lead electrocardiogram (ECG) recording. All the subjects were eligible based on the following criteria: age ≥18 years old, both male and female; male subjects’ weight ≥50 kg, female subjects’ weight ≥45 kg; body mass index between 19 and 26 kg/m^2^. Exclusion criteria included the following: subjects with any significant, acute, chronic, or infectious disease of the respiratory system, circulatory system, cardiovascular system, digestive system, nervous system, etc.; subjects that used concomitant treatments before or during the study period (i.e., being within 4 weeks of exposure to surgery, within 30 days of using any drug that inhibits or induces hepatic metabolizing enzymes, and within 14 days of using any medicines), alcohol abuse, etc.; female subjects who were pregnant, nursing, or rejected using more than one type of adequate contraception. All the eligible subjects were apprised of the risks of the trial and read, understood, and signed the written informed consent forms prior to participation. A subject had to be removed from the study if the subject withdrew the consent, if the investigator considered it appropriate for patient’s safety reasons, or if the subject missed the follow-up.

The entire trial was conducted in accordance with the principles of the Declaration of Helsinki for biomedical research involving human subjects (World Medical Association, 2013), International Conference on Harmonisation Guidelines for Good Clinical Practice (International Conference on Harmonisation of technical requirements for registration of pharmaceuticals for human use, 2001), (China National Medical Products Administration of the People’s Republic of China, 2015), and local regulatory guidelines of the (China National Medical Products Administration of the People’s Republic of China, 2003).

### Medication

Lexapro^®^ containing escitalopram oxalate 10mg tablets (lot no. 2505420, 2532241; expiration date March 2019, December 2019), purchased from H. Lundbeck A/S (Copenhagen, Denmark) were used in trails. Drugs were preserved under the recommended storage conditions.

### Study Design

The data obtained from the results of subjects treated with a reference formulation in two single-center, randomized, open-label, two-period, two-treatment crossover bioavailability studies used the same reference formulation of escitalopram conducted at Changsha Central Hospital, China. In total, 96 healthy adult subjects were enrolled in the study, 48 subjects in each clinical trial. These clinical trials included fasting and fed tests, and both the groups included males and females. The protocols were approved by the Independent Ethics Committee of Changsha Central Hospital. The clinical trial was registered in Chinese Clinical Trial Registry (Registration number: ChiCTR1900027226).

These studies consisted of two periods with a 2 weeks washout interval. All the subjects fulfilling the criteria were randomly stratified by gender to ensure the presence of male and female subjects in each group. Then, the subjects were provided a random number by using a table of random numbers generated by using SAS 9.4 software (SAS Inc., Cary, USA).

Subjects received a single dose of 10 mg escitalopram tablet according to the random numbers within 30 min after a 10 h fasting period or within 30 min after beginning the consumption of a recommended high fat breakfast (150 calories of protein, 250 calories of carbohydrates, 500–600 calories of fat; total calories approximately 800–1,000). Tablets were administered with 240 ml of water to each subject in each period. Drinking was prohibited for 1 h before and after medication. Apart from this, the amount and time of drinking were not strictly controlled. Subjects had unified, standard meals 4 and 10 h after drug administration.

The composition of meal was determined based on the FDA guidelines and consisted of two fried eggs, two fried fritters, and 250 ml of whole milk. The detailed composition and calories of the HF meal used in this study are shown in Supplementary Table S1.

### Safety Assessments

The safety profile and tolerability were assessed throughout by monitoring any adverse events (AEs) at 2, 4, 8, 12, 24, 48, 72, 96, and 120 h after dosing, reported in response to a nonleading question, physical examinations, ECGs, and laboratory (at screening, baseline, and follow-up) and vital signs (predose, 2, 4, 8, 12, 24, 48, 72, 96, and 120 h after dose and follow-up) assessments.

### PK Assessments

Serial blood samples (5 ml) for PK assessment were collected from an indwelling catheter or by direct venipuncture prior to the administration (*t* = 0) and 0.5, 1, 1.5, 2, 3, 4, 5, 6, 7, 8, 12, 24, 48, 72, 96, and 120 h after the administration of escitalopram tablet. Plasma samples were isolated in blood collection tubes by centrifugation (3,500 rpm, 10 min) at 4°C within 1 h after sampling, transferred to labeled storage tubes and stored at −70°C pending workup and analysis.

Descriptive statistics of PK parameters were calculated using established noncompartmental methods, utilizing the WinNonLin version 6.3 software (Pharsight Corporation, USA). The PK parameters determined for each participant included apparent terminal half-life (t_1/2_), maximum plasma concentration (C_max_), time to C_max_ (t_max_), area under the plasma concentration-time curve from the time of administration up to the last time point with a measurable concentration post-dose (AUC_0-t_), AUC extrapolated to infinity (AUC_0-∞_).

### Analytical Methods

All the samples were detected by liquid chromatography (UPLC I-Class, Waters company, USA) -Tandem Mass Spectrometry (Xevo TQ-S, Waters company, USA) (LC-MS/MS) method, validated at Duxact Biotech Co., Ltd. (Changsha, China). A brief description of the method is as follows. The plasma samples were pretreated by protein precipitation method. The internal standard is escitalopram-D4. Using Waters ACQUITYUPLC-HSS T3 1.8 μm (2.1 mm × 50 mm) chromatographic column, 0.2% formic acid aqueous solution (phase A)-acetonitrile (phase B) as the mobile phase, isocratic elution, flow rate 0.4 ml·min^−1^. An electrospray ion source (ESI) and a positive ion multireaction monitoring mode for detection were used. The ion pairs of escitalopram and internal standard escitalopram-D4 are m/z 325.24→m/z109.16 and m/z 329.22→m/z 113.14, respectively. The linear range is 0.100–25.000 ng/ml. The retention time of analyte and internal standard is 0.570 ± 0.1 min. The single injection time is 1.2 min.

It was selective, accurate, and precise. The accuracy ranged from 90.8 to 107.2% of the true value. The intra-batch and inter-batch ranged from 2.0 to 3.2% and from 2.8 to 5.3% of the relative standard deviation (RSD), respectively. The lower limit of detection was 0.1 ng/ml. The calibration curve was linear over the concentration range of 0.2–100 ng/ml, with *r* ^2^ > 0.999. Escitalopram plasma sample could be stored at –70°C for at least 93 days and was stable for more than 50 h when left at room temperature. Escitalopram in plasma was stable after three freeze-thaw cycles. The final processed sample could be left in autosampler at 15°C for at least 24 h. The stock solutions of escitalopram could be kept at –20°C for at least 47 days.

According to this analytical method validation data according to the currently accepted US FDA bioanalytical method validation guidance, this modified method for the determination of escitalopram in plasma was considered reliable and suitable for pharmacokinetic study of escitalopram.

### Genotype of *CYP2C19*

The genotyping was carried out at Duxact Inc. (Changsha, China). CYP2C19*2, *3 and *17 mutations of 90 candidates were determined by the genotyping of TaqMan MGB Probe Method based on a qPCR Platform based on the SNP site rs4244285 (CYP2C19*2), rs4986893 (CYP2C19*3), and rs12248560 (CYP2C19*17). According to the previous studies, the subjects were genotypically classified into the following four groups on the basis of the analysis for CYP2C19: ultrafast metabolizer (UM, CYP2C19*17/*17, CYP2C19*1/*17) group, extensive metabolizer (EM, CYP2C19*1/*1) group, intermediate metabolite (IM, CYP2C19*1/*2, CYP2C19*1/*3) group, poor metabolizer (PM, CYP2C19*2/*2, CYP2C19*3/*3, CYP2C19*2/*3) group (Qiao et al., 2006; Milosavljevic et al., 2021).

### Prediction of Steady-State Blood Drug Concentration

Escitalopram is rapidly and nearly completely absorbed in human (Rao, 2007); In addition, possible confounding factors in this clinic trial, including gender and food, had been excluded. Last, the parameters in the formula are available based on our clinical trials. Therefore, the prediction of average C_ss_ (C_av, ss_) and minimum C_ss_ (C_ss, min_) for different types of metabolizers could be calculated from the PK parameters using Formulas 2–1 and 2–2:

$$C_{av,\;ss}=\frac{F\;\cdot \;D_{M\;}}{CL\cdot \tau \;}$$ (2–1)

$$C_{ss,\;\;min}=\frac{F\;\cdot \;D_{M\;}\cdot \;e^{-k\cdot \tau }}{V\cdot \left(1-e^{-k\cdot \tau }\right)}$$ (2–2)

The prediction of dose was calculated using Formula 2–3:

$$Dose=\;\frac{F}{CL\cdot \tau \cdot C_{av,\;ss}}$$ (2–3)

F, bioavailability; DM, maintenance dose; CL, apparent plasma clearance; V, apparent volume of distribution, k, elimination rate constant; τ, dosing interval.

The WinNonLin version 6.3 software (Pharsight Corporation, USA) was utilized to establish the predicted schematic diagram of multiple doses.

### Statistical Methods

The concentrations of escitalopram were measured as the mean concentration of each time point and standard deviation (SD), except for t_max_, which was expressed as median (range). The major PK parameters such as t_max_, t_1/2_, AUC, and C_max_ under fasting or fed conditions, male or female subjects, and genotypes were statistically analyzed using SAS software (SAS Institute Inc., USA).

Analysis of Variance (ANOVA) was conducted after the natural log-transformation of major PK parameters to evaluate the effects of food, gender, and genotype on the PK of escitalopram.

## Results

### Equivalence Test of Two Clinical Trials

The equivalence test showed no statistical difference in the main PK parameters between trails 1 and 2 (Supplementary Table S2). Although AUC_0-∞_ 90% CI fell in 79–106, it still showed no statistical difference between trail 1 and trail 2. Therefore, the data obtained from these two trails could be combined for the subsequent analysis.

### Demographic Characteristics

In total, 96 healthy subjects were enrolled in this study. All the subjects met the eligibility criteria for the protocol. Six subjects withdraw from the clinical trial; therefore, 90 healthy subjects (67 males and 23 females) were included in the statistical analysis. Baseline demographics across both the groups are shown in Table 1.

**TABLE 1:** Demographic characteristics and Summary statistics for the main pharmacokinetic parameters of study subjects.

Characteristics/Parameters	Fasting (*N* = 45)	Fed (*N* = 45)	Male	Female
			(*N* = 67)	(*N* = 23)
Age (year)	19–38 (25 ± 5)	18–44 (24 ± 6)	18–44 (25 ± 6)	18–33 (23 ± 4)
BMI (kg/m^2^)	19.00–25.60 (22.23 ± 1.90)	19.30–25.00 (21.69 ± 1.55)	19.00–25.60 (22.04 ± 1.73)	19.20–25.50 (21.73 ± 1.81)
t_1/2_ (h)	35.5 ± 11.6	34.6 ± 13.0	36.1 ± 12.8	32.0 ± 10.2
t_max_ (h)	3.0 (1.5–8.0)	4.0 (1.0–12.0)	3.0 (1.0–8.0)	3.0 (1.5–12.0)
C_max_ (ng/ml)	12.5 ± 3.3	12.9 ± 3.0	12.4 ± 3.2	13.6 ± 2.8
AUC_0∼t_ (h*ng/mL)	471.6 ± 159.3	481.6 ± 188.4	469.8 ± 173.8	496.3 ± 175.2
AUC_0∼∞_ (h*ng/mL)	537.5 ± 226.9	554.7 ± 284.7	544.6 ± 265.9	550.6 ± 230.8

Age and BMI expressed as range (mean ± standard), t_max_ expressed as median for range. Other values are presented as mean ± standard deviation. BMI, body mass index; t_1/2_, participant included apparent terminal half-life; C_max_, maximum plasma concentration; t_max_, time to C_max_; AUC_0-t_, area under the plasma concentration-time curve from the time of administration up to the last time point with a measurable concentration post-dose; AUC_0-∞_, AUC extrapolated to infinity.

### Effect of Food

The fasting and fed tests were used to evaluate if food affects the PK parameters of escitalopram. All the available PK parameters recorded for each subject were included in the PK assessments, summary statistics, and statistical analyses, as predefined in the study protocol. The mean plasma concentration-time curves for the fasting and fed tests of escitalopram are shown in Supplementary Figure S1. A summary of the PK parameters of fasting and fed tests of escitalopram is shown in Table 1. The equivalence test showed the ratio 90% CI fell in 80–125%, i.e., the fasting test and fed test were equivalent, indicating that there was no statistical difference between these two treatments (Supplementary Table S2). These results show that food might not affect the PK parameters of escitalopram on Chinese population.

### Effect of Gender

**FIGURE 1:**
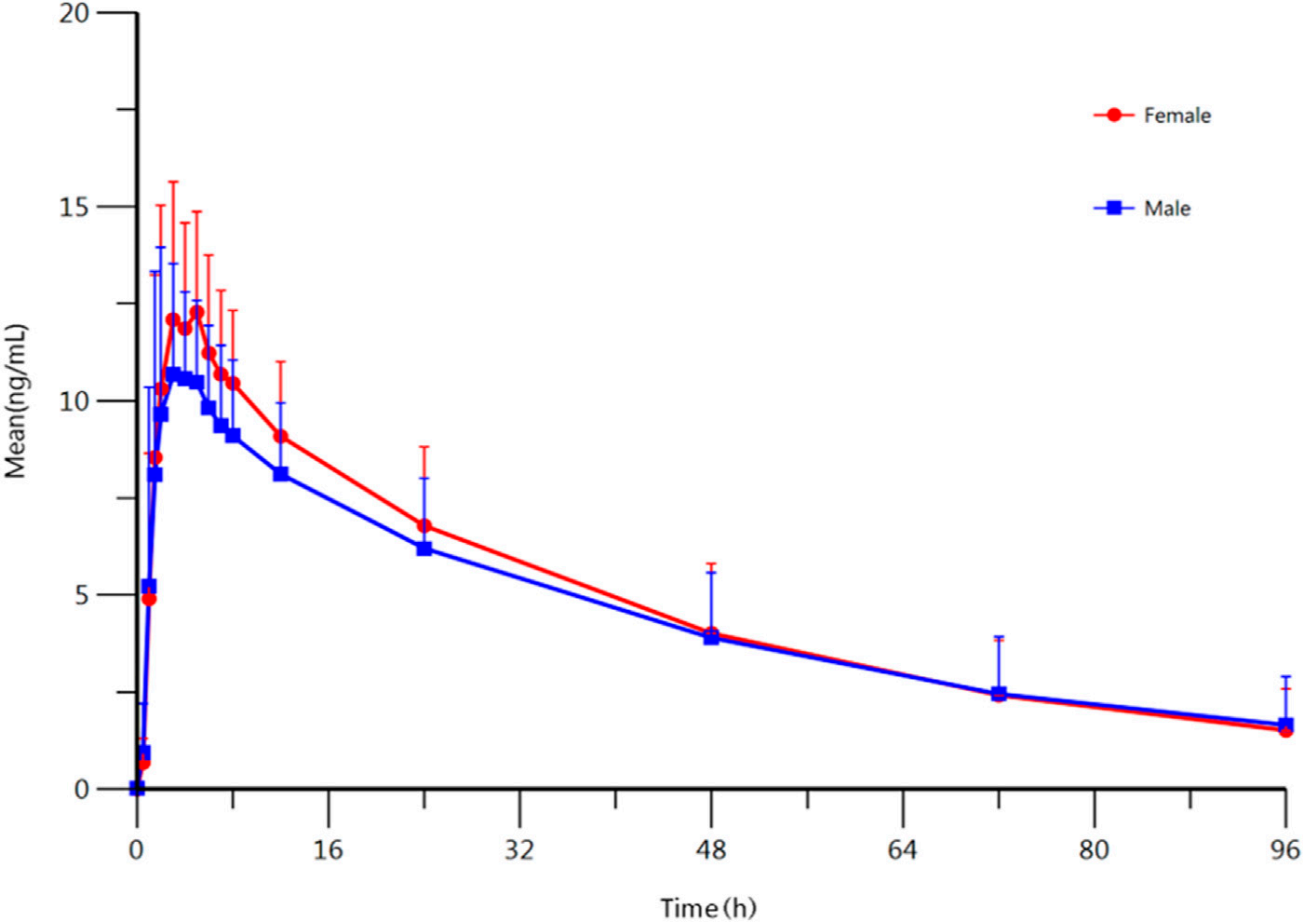
Mean plasma concentration-time profile of escitalopram after oral administration of 10 mg escitalopram tablet in males (*n* = 67) and females (*n* = 23), respectively. All values are presented as mean ± standard deviation.

To confirm the effect of gender, the PK parameters of female and male subjects were also analyzed. The summary of PK parameters of escitalopram for female and male subjects is shown in Table 1. The mean plasma concentration-time curves of escitalopram for different genders are shown in Figure 1. The equivalence test showed that the ratio 90% CI fell in 80–125%, i.e., the main PK parameters of male subjects and female subjects were equivalenced, indicating that there was no statistical difference between these two treatments (Supplementary Table S2). These results show that gender might not influence the PK parameters of escitalopram in Chinese population.

### Effect of Genotype

According to our results, food and gender might not affect the PK parameters of escitalopram; therefore, the PK parameters were analyzed by genotype without considering about food and gender. Out of 90 candidates, the DNA quality of 88 candidates satisfied the sequencing requirements. Among these candidates, 13 subjects were *CYP2C19* PMs with a *CYP2C19* genotype of *2/*2 allele (*n* = 11), *3/*3 allele (*n* = 1), and *2/*3 allele (*n* = 1), 47 subjects were *CYP2C19* IMs with a *CYP2C19* genotype of *1/*2 allele (*n* = 37) and *1/*3 allele (*n* = 10), 37 subjects were *CYP2C19* EMs with a *CYP2C19* of *1/*1 allele (*n* = 39); one subject was *CYP2C19* UM with a *CYP2C19* of *1/*17 allele (*n* = 1). The UM was not included in the analysis due to the lack of candidates. The ANOVA was used to compare the PK parameters in different types of metabolizers.

**FIGURE 2:**
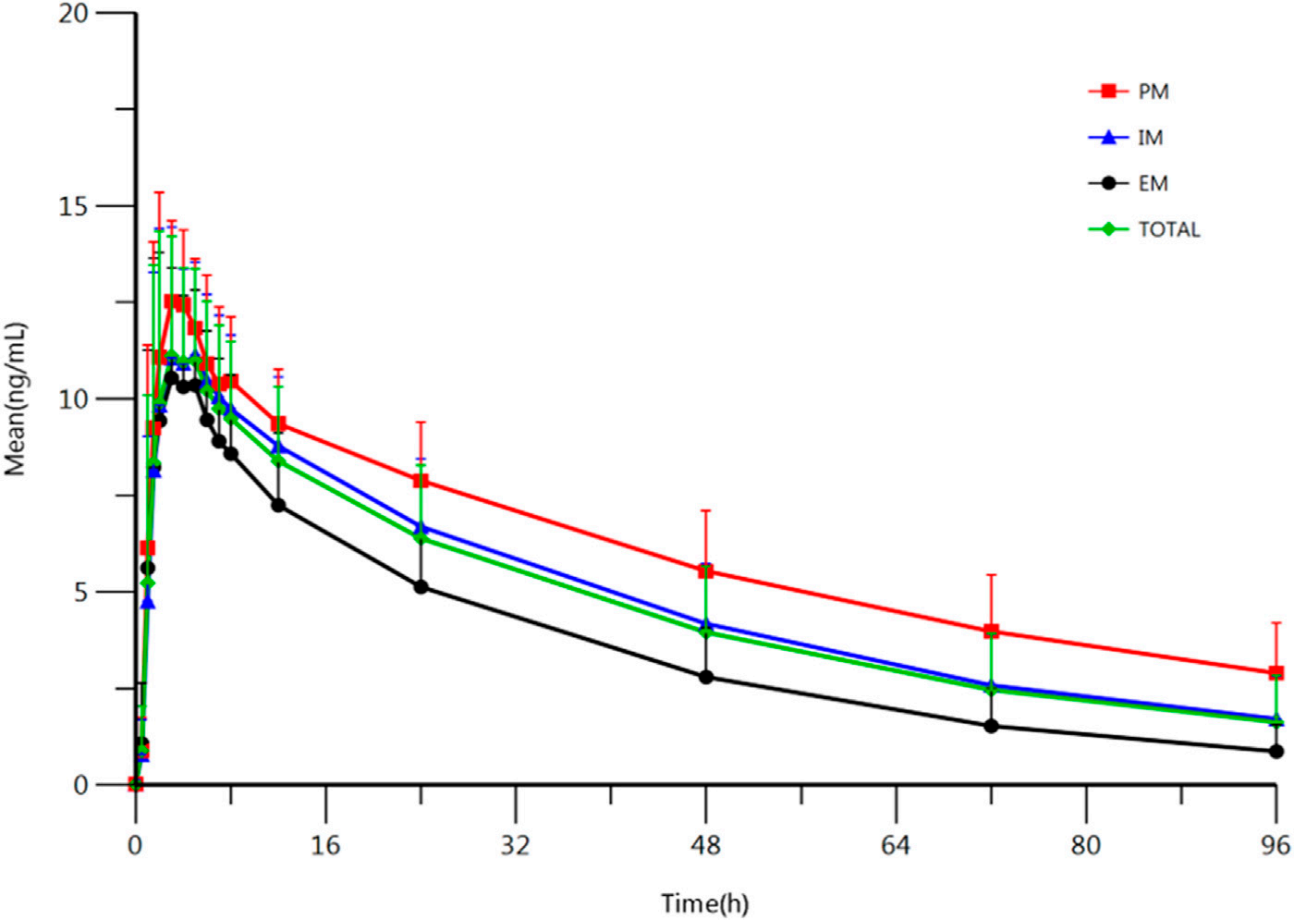
Mean plasma concentration-time profile of escitalopram after oral administration of 10 mg escitalopram tablet in IM (*n* = 47), PM (*n* = 27) and EM (*n* = 13), respectively. All values are presented as mean ± standard deviation.

The results indicate that most of the PK parameters showed statistically difference between different types of metabolizers. AUC_0-t_, AUC_0-∞_, and t_1/2_ were statistically different among those three types of metabolizers: Compared with the EM, the exposure to escitalopram increased by 106% (95%CI, 61–162); t_1/2_ of escitalopram increased by 75% (95%CI, 46–110) in the PM. Compared with the IM, the exposure to escitalopram increased by 41% (95%CI, 13–77); t_1/2_ of escitalopram increased by 32% (95 %CI, 12–56) in the PM. However, C_max_ was not statistically different among the IM, EM, and PM. The PK parameters of IM are closed to the total subjects. The summary of PK parameters of escitalopram for the IM, EM, PM, and all the subjects is shown in Table 2. The PK parameters of IM are similar to the results of all subjects without distinguishing the genotypes. The analysis of PK parameters of the metabolizers is shown in Table 3. The mean plasma concentration-time curves of escitalopram for the three types of metabolizers and total subjects are shown in Figure 2.

**TABLE 2:** Summary statistics for the main pharmacokinetic parameters of different type of metabolizers of escitalopram.

PK parameters	PM (*N* = 13)	IM (*N* = 47)	EM (*N* = 27)	Total (*N* = 87)
t_1/2_ (h)	47.2 ± 10.0	36.4 ± 12.4	27.1 ± 7.3	35.1 ± 12.5
t_max_ (h)	3.0 (1.5–5.0)	3.0 (1.5–12.0)	3.0 (1.0–6.0)	3.0 (1.0–12.0)
C_max_ (ng/ml)	13.8 ± 2.4	12.9 ± 3.0	12.4 ± 3.6	12.9 ± 3.1
AUC_0∼t_ (h*ng/ml)	640.6 ± 160.8	500.0 ± 159.7	366.8 ± 133.9	479.7 ± 175.5
AUC_0∼∞_ (h*ng/ml)	793.9 ± 248.1	574.2 ± 249.5	392.1 ± 164.6	550.5 ± 259.3

Values are presented as mean ± standard deviation. t_max_ expressed as median for range. t_1/2_, participant included apparent terminal half-life; C_max_, maximum plasma concentration; t_max_, time to C_max_; AUC_0-t_, area under the plasma concentration-time curve from the time of administration up to the last time point with a measurable concentration post-dose; AUC_0-∞_, AUC extrapolated to infinity.

**TABLE 3:** Comparison of pharmacokinetic parameters of different type of metabolizers.

PK parameter	IM (*N* = 47)	EM (*N* = 27)	PM (*N* = 13)	PM/EM ratio	PM/EM pValue	PM/EM 95%CI	PM/IM ratio	PM/IM 95%CI	PM/IM pValue
Ln AUC_0-t_ (h*ng/ml)	478.0	347.8	619.7	1.78	<0.001	145–218	1.30	107–157	0.007
Ln AUC_0-∞_ (h*ng/ml)	534.1	366.7	753.9	2.06	<0.001	161–262	1.41	113–177	0.003
Ln C_max_ (ng/ml)	12.5	11.9	13.5	1.13	0.124	97–133	1.08	93–125	0.319

The ANOVA was used in analysis. CI Confidence Interval. C_max_, maximum plasma concentration; AUC_0-t_, area under the plasma concentration-time curve from the time of administration up to the last time point with a measurable concentration post-dose; AUC_0-∞_, AUC extrapolated to infinity.

### Prediction of Blood Drug Concentration of Steady-State

**FIGURE 3:**
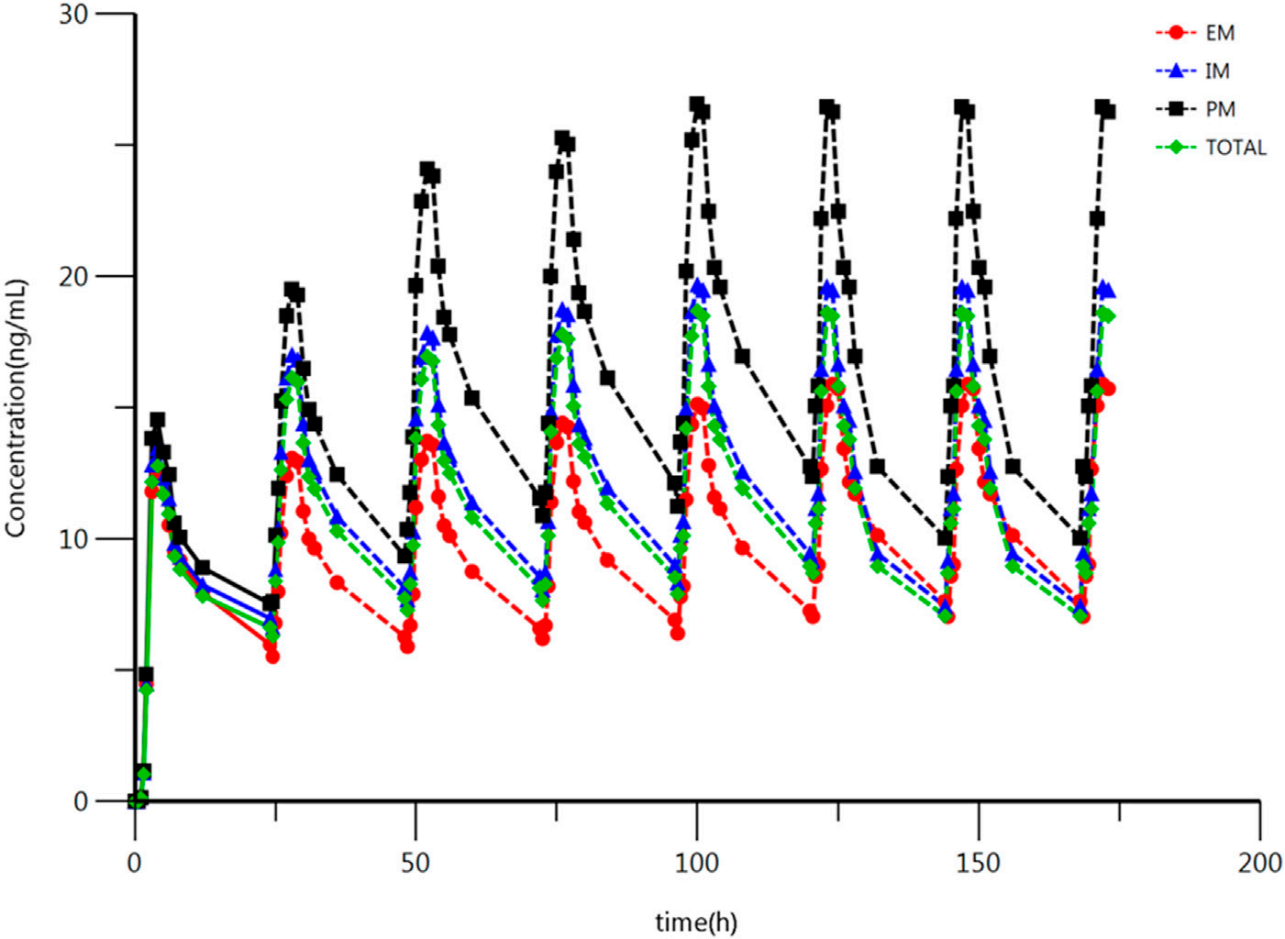
Plasma concentration-time schematic diagram of escitalopram after oral (multi dose) administration of 10 mg escitalopram tablet in the IM, PM and EM. Based on the original concentration data and the prediction of C_ss_. All values are presented as mean ± standard deviation.

The main PK parameters were obtained from these single dose clinical trials. However, the treatment of depression disorder is a long-term process, and escitalopram often has multiple doses in the clinical treatment (Gartlehner et al., 2016). According to previous reports and clinical guideline, 10 mg/d is the common clinical treatment dose, which is the same as the single dose in this study (Hicks et al., 2015; Gartlehner et al., 2016). The bioavailability of escitalopram was reported as 80% (Rao, 2007). Therefore, it is available to predict the C_av, ss_ and C_ss, min_ according to the PK parameters using Formulation 2–1 and 2–2. The summary of C_av, ss_ and C_ss, min_ of escitalopram for the IM, EM, and PM, and all the subjects are shown in Table 4; the prediction of IM is similar to the results of all subjects without distinguishing genotypes. The ANOVA was used to compare the results among different type of metabolizers. The results indicate that C_av, ss_ and C_ss, min_ were statistically different among different types of metabolizer in Chinese population. Compared to the EM, the C_av, ss_ of escitalopram increased by 106% (95 %CI, 61–162); the C_ss, min_ of escitalopram increased by 139% (95% CI, 80–217) in the PM; compared with the IM, the C_av, ss_ of escitalopram increased by 41% (95 %CI, 13–77); the C_ss, min_ of escitalopram increased by 51% (95 %CI, 13–77) in the PM. The results of IM are close to the total subjects. The analysis of C_av, ss_ and C_ss, min_ of different types of metabolizers is shown in Table 5. The predicted schematic diagram of multiple doses to reach the calculated predicted C_ss_ on different metabolizers and total subjects are shown in Figure 3. A preliminary prediction of dose was obtained using Formulation 2–3: The results show that compared with all the subjects, it is necessary to decrease 22% of dose in the PM and increase 59% of dose in the EM.

**TABLE 4:** Summary statistics for the C_ss_ of different type of metabolizers of escitalopram.

PK	PM (*N* = 13)	IM (*N* = 47)	EM (*N* = 27)	Total (*N* = 87)
C_av, ss_ (ng/ml)	26.5 ± 8.3	19.1 ± 8.3	13.1 ± 5.5	18.4 ± 8.6
C_min, ss_ (ng/ml)	22.2 ± 7.7	15.2 ± 7.8	9.5 ± 4.9	14.5 ± 8.1

All values are presented as mean ± standard deviation. C_av, ss_, average steady-state blood concentration; C_ss, min_, minimum steady-state blood concentration.

**TABLE 5:** Comparison of C_SS_ of different type of metabolizers.

PK parameter	IM (*N* = 47)	EM (*N* = 27)	PM (*N* = 13)	PM/EM ratio	PM/EM 95%CI	PM/EM pValue	PM/IM ratio	PM/IM 95%CI	PM/IM pValue
Ln C_av, ss_ (ng/ml)	17.8	12.2	25.1	2.06	161–262	<0.001	1.41	113–177	0.003
Ln C_min_, _ss_ (ng/ml)	13.8	8.7	20.7	2.39	180–317	<0.001	1.51	116–196	0.003

The ANOVA was used in analysis. CI Confidence Interval. C_av, ss_, average steady-state blood concentration; C_ss, min_, minimum steady-state blood concentration.

## Discussion

As an SSRI, escitalopram is widely used in the treatment of depression disorder and anxiety disorder (Yu et al., 2021). Its efficacy and toxicity show variation among different genotypes in previous studies (Chang et al., 2014; Jukić et al., 2018). Although escitalopram has been approved in China for about 15 years, there is a lack of data on the PK parameters of different genotypes in the Chinese population. Therefore, the effect of different genotypes was evaluated in 96 healthy Chinese subjects, and other factors such as food and gender that might alter the PK parameters were also considered in this study. The C_ss_ was predicted by PK parameters using formulas to simulate clinical medication. The results show that food and gender might not affect the PK parameters of escitalopram, and the genotypes might be one of the major factors that affect the PK parameters of escitalopram. The prediction indicates that the PM has increased C_ss_ than the EM and IM, which might provide evidence for the adoption of escitalopram dosage in Chinese population.

During the study, no drug combination occurred in the subjects; therefore, there was no potential effect of drug-drug interactions on PK parameters. The PK parameters showed no statistical difference between the fed and fasting groups, i.e., food might not alter the PK parameters in Chinese population. The results are similar with the results of previous studies in other races (Søgaard et al., 2005; Li et al., 2020; Landy and Estevez, 2021), indicating that escitalopram can be administered with or without meals in Chinese population. Then, the PK parameters showed no statistical difference between male and female, i.e., gender does not affect the PK parameters in Chinese population. The same results were obtained in the studies on other races like Egyptian population and Caucasian population (ElKady et al., 2018; Landy and Estevez, 2021). The *CYP2C19* genotypes might play a key role in the PK parameters of escitalopram: The PM had the highest AUC_0-t_ and AUC_0-∞_, and the longest t_1/2_ than the EM and IM. Different C_max_ levels were found among the three types of phenotypes, but they were not significant, i.e., the degree of *CYP2C19* genotype effects on C_max_ might not be as deep as other PK parameters. The results of AUC show that the PM might have higher exposure than EM in Chinese population, which might affect the efficacy and safety of escitalopram treatment. The same results were reported in other populations, although the specific PK parameters were not exactly the same: The effects of individual variation on the efficacy and toxicity of different *CYP2C19* genotypes were reported in patients of Caucasian population and Brazilian population (Chang et al., 2014; Jukić et al., 2018; Bernini de Brito and Ghedini, 2020; Landy and Estevez, 2021; Milosavljevic et al., 2021). Because of a higher mutation rate of the PM *CYP2C19* genotype in Chinese population, the association between metabolizers and individual variations should be considered in the treatment of escitalopram in Chinese population (Dorji et al., 2019; Zhou et al., 2019). However, escitalopram often has multiple doses in the clinical treatment, and C_ss_ is a key factor reflecting the status of clinical medication (Landy and Estevez, 2021). The common clinical dosing regimen of escitalopram and dosing interval are used to predict the C_av, ss_ and C_ss, min_ of different types of metabolizers. Although C_max_ showed no statistical difference in single dose results, the prediction showed that compared with the EM, the C_av, ss_ and C_ss, min_ of escitalopram increased by 106 and 139% in the PM, respectively. The increased C_ss, min_ indicates the rising risk of side effects in the treatment of escitalopram. The therapeutic window of escitalopram is still not clear enough. Most of the studies focus on the pharmacokinetics of escitalopram did not provide the C_ss_ of escitalopram. From the existing reports: the rational C_ss_ of escitalopram (10 mg/d) in humans are from 8 to 52 ng/ml, which were consistent with the results in this study (Rao, 2007; Chang et al., 2014; Jukić et al., 2018). The results indicate that the genetic testing of *CYP2C19* genotype is necessary before the treatment of escitalopram in Chinese population. The same recommendations were provided by the studies in Caucasian population according to the Clinical Pharmacogenetics Implementation Consortium (Hicks et al., 2015). The preliminary prediction showed that compared with all the subjects, it is necessary to decrease 22% of dose in the PM and increase 59% of dose in the EM. However, the initial therapy dose and the reducing dosage of escitalopram in Chinese population need to be investigated in the follow-up research.

Some limitations of this study are as follows: First, the unavailability of UM data was attributed to the limited simple size and the low gene frequency in Chinese population (Dorji et al., 2019). Secondly, the effect of age was not considered in our study, although it was reported to be associated with the PK parameters of escitalopram (Waade et al., 2014; Landy and Estevez, 2021). Thirdly, the clinical trials were only conducted in healthy populations, without considering the changes in PK under disease states and the efficacy of escitalopram. Lastly, the C_ss_ was preliminarily predicted using the PK formula which may not fully represent the universal data. In future studies, it is necessary to conduct clinical trials considering a sufficient number of subjects for each genotype and the influence of age and disease state in Chinese population. The clinical trials enrolling patients to investigate the efficacy and toxicity of escitalopram of different metabolizers in Chinese population should also be considered in the future. In addition, the clinical trials with multiple doses of escitalopram should also be considered to assess the certain C_ss_ data in Chinese population in follow-up research.

In summary, the PK parameters in Chinese population obtained in our study might help to determine the clinical dosing regimen and design follow-up studies. The intraindividual variation of exposure of escitalopram was 30.92%, providing a basis to be used in sample size estimations of Chinese population in future clinical trials. The PK parameters showed that the exposure of escitalopram was higher in the PMs in Chinese population. These results indicate that a decreased dose of escitalopram in the PM on Chinese population should be considered due to the higher frequency of PM in Chinese population than that in Caucasian populations (Myrand et al., 2008). The prediction that the C_ss_ in different metabolizers may be different suggests that genotype might be a prerequisite for determining the dosage of escitalopram in Chinese population. Further research should be conducted to establish a guideline for the dosing of *CYP2C19* genotypes in Chinese population as in Caucasian populations (Hicks et al., 2015). Our results suggested that to decrease the toxicity and increase the efficacy of escitalopram in Chinese population, genetic testing before medication, adjustment of dose, and therapeutic drug monitoring should be considered in the clinical treatment of escitalopram. The results indicated CYP2C19 genotyping may provide a better treatment strategy when administering escitalopram in Chinese population, which will be beneficial for a safer and more effective application of escitalopram in the Chinese population.

## Conclusion

Based on the results of this study, in Chinese population, food and gender might not affect the PK parameters of escitalopram, while *CYP2C19* might affect it: The PM might have a higher exposure and t_1/2_ than the EM and IM. The prediction of C_ss_ indicates that PM might have a higher plasma concentration of escitalopram, which reminds us that genetic testing should be conducted before the medication, and the adjustment of escitalopram in the PM should be considered in the clinical treatments in Chinese population.

## Data Availability

The raw data supporting the conclusions of this article will be made available by the authors, without undue reservation.
