# Supplementary material for: CYP2C19 Genotyping May Provide a Better Treatment Strategy when Administering Escitalopram in Chinese Population
Source: Front Pharmacol. 2021 Aug 27;12:730461. doi: 10.3389/fphar.2021.730461 (PMC8429954; doi:10.3389/fphar.2021.730461)
Supplement: Supplementary file 2 [file DataSheet2.docx]

Supplementary Material

**Supplementary Table 1. Compositions of the high fat meal.**

| **Nutrient content** | **Protein (g)** | **Carbohydrates (g)** | **Fat (g)** | **Total (g)** |
| --- | --- | --- | --- | --- |
| Two fried fritters (100g) | 6.9 | 50.1 | 17.6 | 74.6 |
| Two fried egg  (100g) | 13.3 | 2.8 | 8.8 | 24.9 |
| Blend Oil  (30g) | 0 | 0 | 30 | 30 |
| Whole milk (250ml) | 0 | 12.5 | 11 | 32.5 |
| Total (g) | 29.2 | 65.4 | 67.4 | 162 |
| Caloric values (KCal) | 116.8 | 261.6 | 606.6 | 985 |
| The proportion of total calories (%) | 11.9 | 26.6 | 61.6 | 100.0 |

The detailed composition and calories of the high fat meal used in the study. 1 g protein calories count by 4 cal; 1 g carbohydrate calories count by 4 cal; 1 g fat calories count by 9 cal.

**Supplementary Table 2. The equivalence test of two clinical trials.**

| **PK** | **Trial2**  **(N=45)** | **Trial1**  **(N=45)** | **Ratio** | **90% CI** |
| --- | --- | --- | --- | --- |
| Ln C_max_（ng/mL） | 11.9 | 12.9 | 0.92 | 85 ~ 101 |
| Ln AUC_0-t_（h*ng/mL） | 433.4 | 462.8 | 0.94 | 83 ~ 106 |
| Ln AUC_0-∞_（h*ng/mL） | 475.7 | 519.9 | 0.92 | 79 ~ 106 |
| Ln t_1/2_（h） | 32.0 | 34.5 | 0.93 | 83 ~ 104 |

Trial 2/Trial 1 Ratio 90% CI fall in 80%~125% means the Trial1 and the Trial2 are equivalence.

t_1/2_, participant included apparent terminal half-life; C_max_, maximum plasma concentration; AUC_0-t_, area under the plasma concentration-time curve from the time of administration up to the last time point with a measurable concentration post-dose; AUC_0-∞_, AUC extrapolated to infinity.

**Supplementary Table 3. The equivalence test of fed and fasting conditions.**

| **Parameters** | **Fed**  **(N = 45)** | **Fasting**  **(N = 45)** | **Fed / Fasting ratio** | **90%CI** | **Lower_pValue** | **Upper_pValue** |
| --- | --- | --- | --- | --- | --- | --- |
| In C_max_ (ng/mL) | 12.6 | 12.1 | 1.04 | 96-113 | <0.001 | <0.001 |
| In AUC_0~t_ (h*ng/mL) | 449.6 | 446.0 | 1.01 | 89-114 | 0.001 | 0.003 |
| In AUC_0~∞_ (h*ng/mL) | 498.8 | 495.8 | 1.01 | 87-117 | 0.006 | 0.009 |

The Fed / Fasting ratio 90%CI fell in 80-125% means the fed treatment and the fasting treatment are equivalence.

C_max_, maximum plasma concentration; AUC_0-t_, area under the plasma concentration-time curve from the time of administration up to the last time point with a measurable concentration post-dose; AUC_0-∞_, AUC extrapolated to infinity.

| **Parameters** | **Female**  **(N = 23)** | **Male**  **(N = 67)** | **Female/male ratio** | **90% CI** | **Lower_pValue** | **Upper_pValue** |
| --- | --- | --- | --- | --- | --- | --- |
| Ln C_max_ (ng/mL) | 13.3 | 12.1 | 1.11 | 100-122 | <0.001 | 0.019 |
| Ln AUC_0~t_ (h*ng/mL) | 468.8 | 440.9 | 1.06 | 92-123 | <0.001 | 0.031 |
| Ln AUC_0~∞_ (h*ng/mL) | 509.3 | 493.2 | 1.03 | 87-123 | <0.001 | 0.034 |

**Supplementary Table 4. The equivalence test of females and males.**

Female/male ratio 90% CI fell in 80%~125% means the female subjects and the male subjects are equivalence.

C_max_, maximum plasma concentration; AUC_0-t_, area under the plasma concentration-time curve from the time of administration up to the last time point with a measurable concentration post-dose; AUC_0-∞_, AUC extrapolated to infinity.


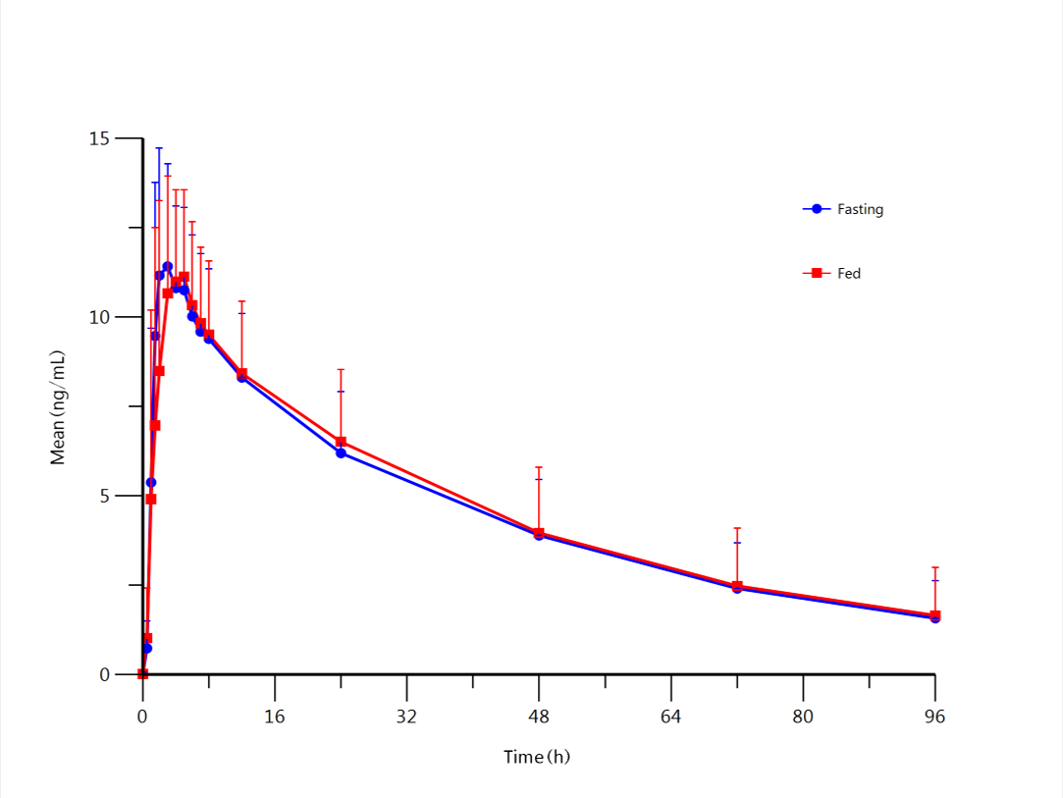


**Supplementary Figure 1. Mean plasma concentration-time profile of escitalopram after oral administration of 10 mg escitalopram tablet in healthy subjects under fasting and fed, respectively.**

There were 45 subjects for each group. All values are presented as mean ± standard deviation.
